# Processes of a Transformation of Young Drivers’ Responsibility for Health—*Carpe Diem*

**DOI:** 10.3390/ijerph18073634

**Published:** 2021-03-31

**Authors:** Agnieszka Kulik, Natalia Kajka, Monika Dacka

**Affiliations:** 1 Department of Psychotherapy and Health Psychology, Institute of Psychology at the Faculty of Social Sciences, The John Paul II Catholic University of Lublin, Al. Racławickie 14, 20-950 Lublin, Poland; agnieszka.kulik@kul.lublin.pl; 2 Department of Developmental Psychology, Institute of Psychology at the Faculty of Social Sciences, The John Paul II Catholic University of Lublin, Al. Racławickie 14, 20-950 Lublin, Poland; monikadacka@kul.lublin.pl

**Keywords:** responsibility for health, young adults, risky driving, health, hedonism

## Abstract

Research most often deals with the relationship between risky behaviour on the road and other aspects of young adults’ lifestyle. It is rare that the sense of responsibility for one’s own life and health and for that of other people on the road is understood, due to the limitation of perceptual data. In this study, we researched 198 young adults (M = 19.75; SD = 1.11) using the Multidimensional Health Locus of Control, the Inventory of Health Behaviours, the Criteria of Health List and a student health survey. The chance of risky driving will increase by 50.7% among young adults as their understanding of health as a state increases. On the other hand, when young people feel that others are responsible for their lives, the likelihood of risky driving will drop by 6.4%. The hedonistic life orientation of a young adult had a significant impact on the results obtained which was connected with their understanding of health.

## 1. Introduction

Risky behaviours on the road are treated differently from other risky behaviours related to health because their assessment is relative and socially subjective, and dependant on time and place (rally driver or professional driver). According to the data published by the Police Headquarters Road Traffic Office, the fatality rate per 100 accidents in Poland is the highest among EU countries and has been on the same level for years [1]. In Poland in the years 2015–2018, young drivers were involved in 22.7–24.9% of all accidents, due to the failure to adjust their speed to traffic conditions. These were people aged 18–24 years, who had had a driving license for no longer than 2 years. As a result, 1916 people were killed and 29,302 were injured. Peers are very often the victims of these accidents. Most car accidents, in which there are injuries, occur on holidays [1,2,3,4,5]. The data from 2016 shows that in the USA, the rate of engaging in risky driving behaviour in this group is as high as 88%. Risky behaviour on the road is also partially attributed to people without a driving licence. In Australian studies, 12% of people aged 17–19 years turned out to be drivers without a licence [6].

### 1.1. Factors Influencing Risky Driving

Although factors influencing dangerous driving have changed throughout the history of motorisation, it is still valid to say that young drivers pose the greatest risk to road safety [7]. Among them, it is men in particular who most often engage in risky driving, but there is an upward trend for young women [3,8]. It has been observed that persons who exceed the speed limit are also more likely to commit other road offences. The way they drive a vehicle depends on subjective factors such as motivation, attitudes, or the perception and acceptance of risk as well as the technical condition of the vehicle and the likelihood of receiving a ticket [5]. The main risk factors also include age and gender [7,8], lack of experience, low level driving skills, lifestyle, and inadequate assessment of situations on the road including one’s ability to face such situations [3]. Accidents are most common among people with a reduced need for stimulation who cannot cope with excessive stress, but also among those with an intensified aggression syndrome [9], as well as driving a vehicle in a negative mood [6]. It has also been observed that people with increased psychoticism, people who seek thrills, who have a preference for instrumental values, and in particular a preference for power (understood as the need to maintain a positive image in the eyes of others) are prone to engaging in dangerous behaviours on the road [8].

There are three types of dangerous behaviours on the road: a tendency to take risks, task-focused orientation, and domination on the road [8]. A characteristic feature of the risk-taking tendency is inconsistency in behaviour, expressed in hasty and random decisions which result in mistakes while driving a vehicle. A task-focused pattern of dangerous behaviour is characterised by the quick and effective achievement of the goal through reluctance towards social norms and a preference for instrumental values. This is accompanied by drivers’ orientation towards hedonism in everyday life which is mainly expressed on the road in fast and overconfident driving and exceeding speed limits. Orientation towards domination on the road manifests itself in the need to subordinate other road users to oneself.

### 1.2. Understanding Responsibility for Health

According to Suchocka [10], the key to being responsible for health is treating it as an important value. Based on research conducted around the world, it has been noticed that young adults do not consider health as an important value [11] or rank it second in their hierarchy [12]. This state of affairs may translate into the negative behaviour young people undertake. Jaworski et al. [13] indicate that the construct of responsibility for health is divided into active involvement and adequate behaviour. Active involvement is understood as the cognitive and motivational aspect of health-promoting activities. Adequate behaviour, in turn, relates to actions taken to maintain or improve health.

Active involvement refers to how health is understood. Research by Stawarz et al. [14] showed that young adults most often perceive it as a *property* and least as a *result*. Being healthy means not having physical ailments and having an optimally functioning body [15]. This manner of understanding health only emphasises the physical aspect, while the perception of health, as a result, is related to concern for adequate nutrition, rest, and avoiding stimulants. Global studies indicate that 12.4% of people aged 15 years and over have insufficient health knowledge, and almost half of the respondents (47.6%) believe that they have insufficient health skills. These results are worrying because lower health skills are typical of people who show poor health, have poorer self-control behaviours, and are less involved in health-promoting activities [16]. When analysing the self-evaluation of the health condition of young people in Poland, it can be noted that it is worse in girls and clearly worsens with age [17]. Health reports from different continents reveal that young people do not always have the information they need to take responsibility for their health [18], although a high level of knowledge is not always a protective factor [19].

Young adults have knowledge about the negative impact of risky behaviours and, at the same time, they are fully aware of the fact that they underestimate the possible consequences [20]. The research by Andruszkiewicz et al. [21] shows that highly motivated people have a significantly higher global sense of understandability of the rationale for limiting health risk activities than people with low motivation. The driving force behind taking specific actions may be the belief in exercising control [22]. On the other hand, people with an external sense of control tend to see the causes of events outside themselves (other people or chance) [23]. The research of Pirzadeh et al. [23] shows that university students achieve high results in terms of the sense of internal control of their health. They seldom believe that their beliefs about chance, luck, or fate can influence their health as effectively as their personal behavioural. When analyzing the health locus of control among university students, some discrepancies in the literature can be seen. Some data indicate gender differences in the intensity of this variable [24], while others do not [23]. In light of the literature on the subject, the role of the locus of health control in the regulation of health behaviours is not clear [22]. Some studies (e.g., [25]) showed that young adults with a stronger sense of inner control more often pay attention to healthy eating and physical activity. There is also no correlation between risky behaviours (i.e., smoking and drinking alcohol) and the locus of health control. It is also recognised that health development is time-sensitive and depends on the social fabric of the environment. This statement is especially true in the period of emerging adulthood, where the interaction between age, personal development, and the environment (peers, school, and social institutions) are very intense. With age, the level of control and resistance to peer group influences increases [26,27]. People become more responsible for their own decisions and actions, including concern for health.

The aim of the current research was to determine the role of responsibility for health in predicting risky driving style in a group of young adults. Generally, this style can be assessed from the subjective (global self-assessment) and objective (by the intensity of indicators) perspectives. In this study, fast driving was understood as the subjectively perceived awareness of their risky behaviour on the road as a driver. It was identified based on the statement: “I try to refrain from driving cars or other vehicles fast” (question 12 from the questionnaire). Indicators of health responsibility are the understanding of health, locus of health control, and health behaviour.

## 2. Materials and Methods

### Description of the Group

The “Students’ Health Behaviours” research project was carried out at universities in the Voivodeship region in Poland by Autor et al. The participants voluntarily took part in questionnaire studies on the determinants of student health behaviours. The project was approved in its entirety by the Ethics Committee of the Institute of Psychology at The John Paul II Catholic University of Lublin (no. 4/08.02.2018 and 5/08.02.2018). The research was carried out during the summer semester of the 2018 academic year. It involved 198 young adults studying in various fields (psychology, landscape architecture, biotechnology, dietetics, romance philology, German philology, dental hygiene, cognitive science, educational sciences, nursing, and law) who participated in the research. The participants included 48 men (24.2%) and 150 women (75.8%) with a mean age of 20 years (M = 19.75; SD = 1.11). Table 1 shows most of the survey participants came from rural areas (35.9%) and large cities (22.7%). More than half of the respondents assessed their health condition as good (50.5%), and 4.5% described their health as very bad. Participants were aware of the presence of chronic diseases in their family of origin (32.3%) as well as their absence (48.5%). Among the respondents, 117 people claimed they refrained from dangerous driving (60 women and 21 men) and 81 people did not (90 women and 27 men).

Analysis of the decision tree allowed us to additionally distinguish three groups of respondents. It will be analysed later in the article, separately for women, men, and a mixed group (women and men). Quantitative and qualitative analysis was used to better explore the transformation of young drivers’ responsibility. In the first stage, quantitative data analysis was used—binary logistic regression analysis. In the second stage, the Process Transformation Reconstruction (*PTR Strategy*) was used. A description of this method will be given below. Quantitative analyses made it possible to identify factors responsible for young adults engaging in risky driving. Qualitative analyses made it possible to determine the diversification of the transformation of responsibility for one’s health in young drivers.

**Figure 1:**
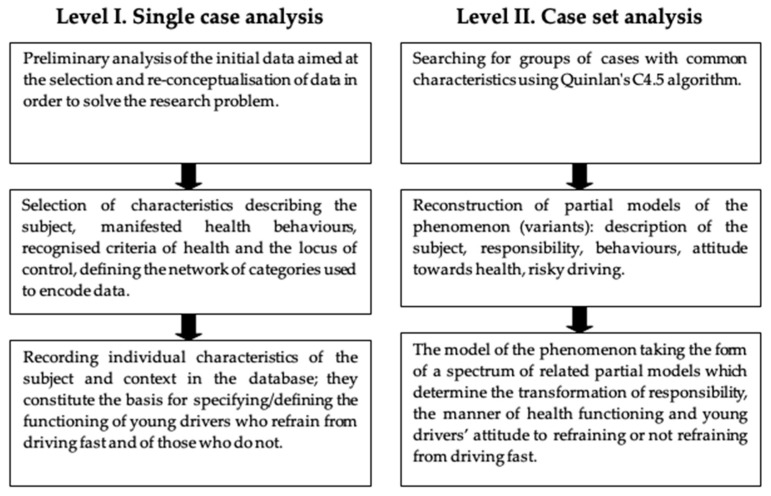
Data analysis scheme: The Process Transformation Reconstruction (PTR) Strategy (a modified version).

The PTR strategy is a complex, qualitative analysis of empirical data, which allows for carrying out research when knowledge of the phenomenon in question is incomplete and when there are difficulties with fully conceptualising the research problem. The aim of the strategy is to reconstruct the course of the phenomenon in terms of its variability and diversity (obtaining new data absent in previous studies) while maintaining the initial properties of the studied subjects at each stage [28]. The PTR strategy is realised on two levels: Level 1—single case analysis (aim: identifying significant characteristics that reflect the participants’ individual life paths), and Level 2—case set analysis (aim: distinguishing sets of cases with similar characteristics on the basis of a decision tree analysis, generated by Quinlan’s C4.5 algorithm [28], a data mining method of symbolic data classification, see Figure 1). This algorithm is one of the methods used in artificial intelligence, based on the mechanisms of generating decision trees, and it is also used in psychological research [29]. They are extremely useful, especially in the study of problems that are sometimes characterised by a high degree of ambiguity and the presentation of their solutions, only in the form of mathematical models, becomes insufficient [30,31]. The model of the phenomenon is assessed based on the accuracy of its predictions [32].

At Level 1, a set of descriptive items and categories was selected for each participant. The selected data from the research methods constituted a set of information allowing for the reconstruction of an individual portrait of each of the respondents in terms of manifested health behaviours, recognised criteria of health, and the locus of control. The next steps involved preliminary data analysis, selection of characteristics describing the subject and the way they function, as well as recording individual characteristics in the database. The consecutive stages of Level I are characterised below.

1. Preliminary analysis of initial data. Preliminary analysis of the results obtained by each of the respondents aims to identify, on the basis of methods (their items) and survey data, categories important for the reconstruction of the characteristics of the respondents, including: manifested health behaviours, recognised criteria of health, and the sense of locus of control. The research used information obtained from three questionnaires: the Inventory of Health Behaviours, the Multidimensional Health Locus of Control, and the Criteria of Health List. The Inventory of Health Behaviours (IHB, Z. Juczyński) consists of 24 items describing various types of behaviour related to health. The tested person responds to each statement on a scale from 1—almost never—to 5—almost always. The second questionnaire used in the study was the Multidimensional Health Locus of Control (MHLC, Z. Juczyński). The scale consists of 18 statements concerning the locus of health control in three aspects: the internal locus, the influence of others, and chance. The examined person responds to the statements on a scale from 1 (I strongly disagree) to 6 (I strongly agree). The last questionnaire used was the Criteria of Health List (LHC, Z. Juczyński), consisting of 24 statements describing various dimensions of physical, mental, and social health.
2. Selection of descriptive categories. As a result of the analysis of the content of items from the methods and the questionnaires, characteristics were selected that allowed the authors to describe each of the respondents. Example: Manifestations of concern for one’s own health—health behaviours (IHB, Item 10: “I follow medical recommendations resulting from my medical examination”).
3. Collating each individual’s characteristics in an individual database. The record created was a kind of basic report describing a person’s features and functioning in terms of health, health attribution, and attitude to their own health.

On the level of case analysis, preserving the value (trustworthiness) of the study involved processing the results (coding, structuring, and decoding) by independent, competent judges.

Level II: case set analysis. At *Level II*, the analysis was focused on identifying study groups with common characteristics. The analysis of the decision tree was the basis for identifying variants (i.e., ways of functioning of subjects in terms of undertaking risky behaviours—risky driving—and responsibility for health recognised as the adopted criteria of health, locus of the sense of health control, and undertaken health behaviours), and subsequently, for ordering them in a manner that reflected differentiation and internal organisation. Detailed analysis of the internal structure of each of the variants and their connections was the basis for building more general structures called sub-models that accurately reflected the transformation of young drivers’ responsibility. The analysis of connections between sub-models was the basis for building a hypothetical model of change in the responsibility of young people for engaging in risky behaviours related to fast driving.

In the subsequent steps, a set of cases with common features was identified, partial models of the phenomenon were reconstructed, and a hypothetical model of the transformation of the phenomenon was developed.

1. The database and distinguishing case sets with shared characteristics.

Case sets with shared characteristics were distinguished using Quinlan’s C4.5 inductive algorithm [33]. The researcher’s task was to prepare a database (in this case: 198 people described with the use of 73 attributes corresponding to personal characteristics, health behaviours, locus of control, criteria of health, and tendencies to engage in risky behaviours—fast driving). At this stage, it was important that the programme posed and verified hypotheses, due to the classification criterion chosen by the researcher: “I try to refrain from driving cars or other vehicles fast” (question 12 from the questionnaire).

2. The decision tree and reconstruction of the partial models of the phenomenon.

The set of qualitative characteristics, derived on the basis of the structure of the decision tree, was then supplemented with data from the databases. The analysis of the decision tree structure was a starting point for the reconstruction of partial models (hereinafter referred to as variants), reflecting the ways of transforming responsibility for one’s own health by young drivers who engage in fast driving or refrain from it.

3. Constructing the phenomenon’s hypothetical model of transformation.

In the PTR strategy, the phenomenon was defined as a process of transformation that reflected partial models (variants). The detailed analysis of the internal structure of each of the variants and their genetic ties served as the starting point for building more general structures (sub-models), which reflected the nature of the analysed phenomenon more broadly. The analysis of relationships between sub-models was the basis for building a hypothetical model of the transformation of responsibility of young drivers who engage in fast driving or refrain from it.

On the level of the case set analysis, the value was maintained by (1) calculating the error rate of the decision tree (here: 1% with the permitted threshold of 25% for incorrect qualifications) and (2) the internal consistency of (a) each of the partial health behaviour patterns and (b) the model’s organisation as a whole.

## 3. Results

Twelve indicators of responsibility for health as well as the gender of the respondents were introduced into the tested logistic regression analysis model (Table 2). The collinearity tests performed for all elements tested in the model range from 1.125 to 1.821 and the tolerance coefficient for these elements ranged from 0.549 to 0.880.

The conducted calculations show that only two factors significantly explained the tendency for risky driving in young adults. These were understanding health as a state and the impact of other important people on controlling young adults’ health. The test of goodness of fit of the data with the Hosmer and Lemeshow model indicated that they were well fitted (χ^2^ (8) = 4.07; *p* = 0.851). Understanding health as a state and the impact of the control other people exerted over the health of young adults made it possible to predict risky driving in young adults (χ^2^ (13) = 22.351; *p* = 0.05) and explained 14.4% of the variance in the dependent variable. The obtained results allowed for the formulation of the following predictions: as the understanding of health as a state increases by one standard deviation unit, the value of the risky driving coefficient increases 1.5 times. This indicates that the chance of risky driving will increase by more than half (50.7%) among young adults when their understanding of health as a state increases by one unit on the scale. On the other hand, the value of the coefficient of risky driving (Exp (B) = 0.936) decreases when young adults assess that the influence of others on their health is lower by one standard deviation unit. As a result, the chances of risky driving in young adults dropped by 6.4%.

**Figure 2:**
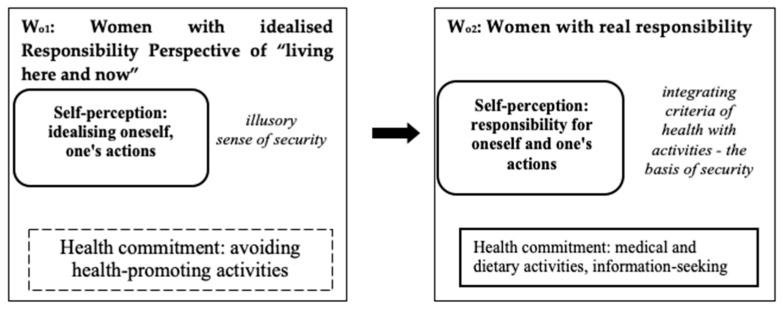
Two sub-models (Wo_1_, Wo_2_) that illustrate the transformation of responsibility for one’s health: from idealized responsibility to real responsibility.

**Figure 3:**
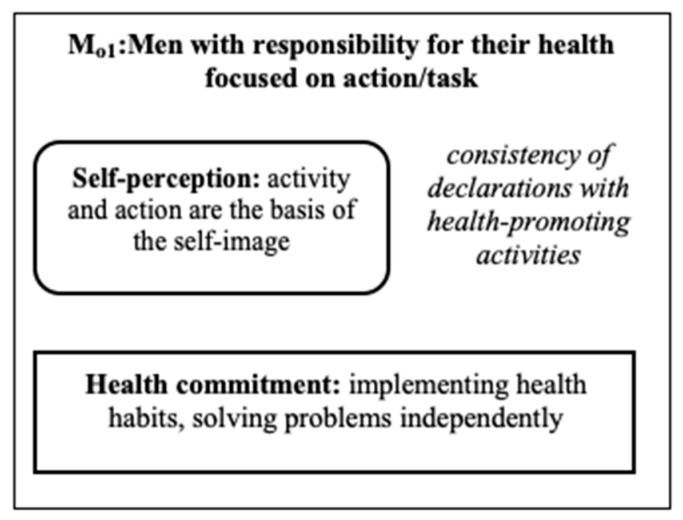
The sub-model of responsibility for their own health (Mo_1_) among men.

**Figure 4:**
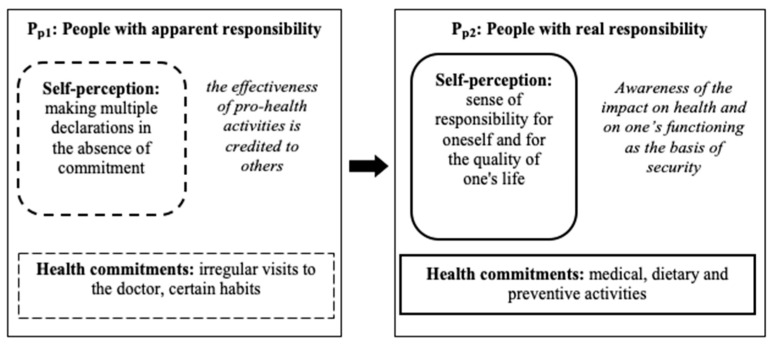
Two sub-models (Pp1, Pp2) showing the transformation of responsibility for one’s health from apparent responsibility to real responsibility.

**Figure 5:**
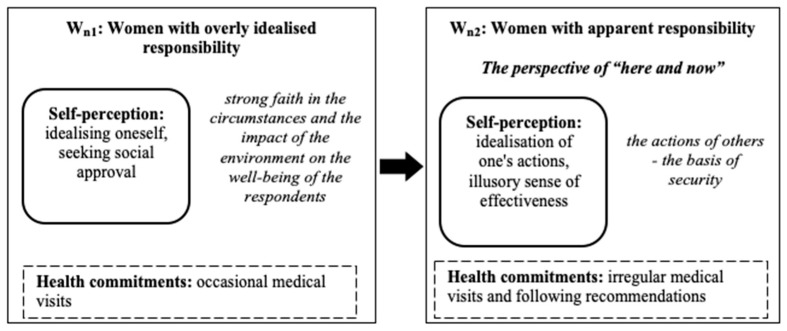
The two sub-models (W_n1_, W_n2_) were distinguished which show the transformation of responsibility for one’s own health from excessively idealised responsibility to apparent responsibility.

**Figure 6:**
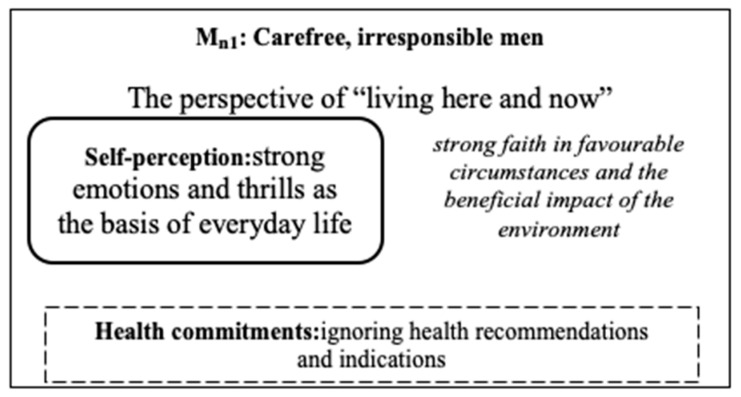
The sub-model: men who are carefree and irresponsible with their health.

**Figure 7:**
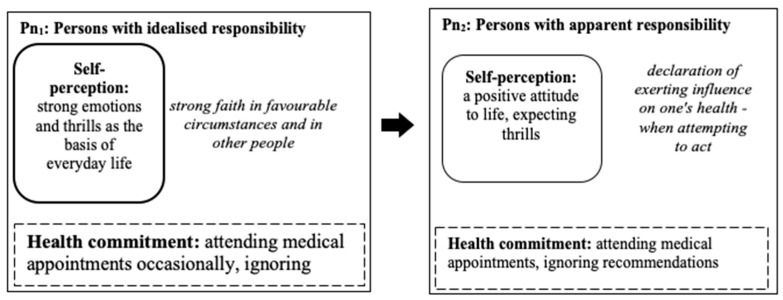
The sub-models responsibility for one’s health from idealised responsibility to apparent responsibility among the mixed group.

In order to better understand the changes in young drivers’ concept of responsibility for health, data analysis was carried out using the PTR Strategy. On that basis, specific sub-models were identified that reflected the transformation of young drivers’ responsibility. The Quinlan C4.5 algorithm used for constructing the decision tree allowed the researchers to correctly determine the prediction of factors that affect engaging in fast driving or refraining from it. The obtained error rate was 1% with the permitted threshold of 25% (it proved a very good accuracy of data classification). The analysis of the decision tree additionally allowed the authors to distinguish three groups of respondents: women, men, and a mixed group. The descriptions of the respondents took into account manifestations of health behaviours, a sense of control and attitude to the criteria of health they recognised. In the first part, the results of people who refrain from driving fast were presented (see Figure 2, Figure 3 and Figure 4), while the second part presented the results of people who do not refrain from driving fast (see Figure 5, Figure 6 and Figure 7).

### 3.1. People Who Refrain from Driving Fast

In the group of women, two sub-models (W_o1_, W_o2_) were selected which illustrated the transformation of responsibility for one’s health: from idealised responsibility to real responsibility.

### 3.2. W_o1_: Women with Idealised Responsibility for Their Health

The sub-model consisted of fifteen women who overly idealised their image of themselves (a responsible person and disciplined in care for their health) or their actions (regular medical visits and compliance with dietary recommendations). The respondents were convinced of the possibility of exerting influence on their own health, but they avoided real actions in situations of necessary health interventions. These women had a hedonistic attitude toward life, they did not notice problems or stressful situations in their daily lives. They were characterised by idealised responsibility for their well-being.

### 3.3. W_o2_: Women with Real Responsibility for Their Health

The sub-model consisted of fifteen women taking real medical and dietary measures in a situation of threat to their health. Thus, the respondents implemented the criteria of health and they recognised and perceived their own and others’ commitment to returning to full strength in the event of an illness. They regularly looked for information about the possibilities of protecting their well-being and the possibility of maintaining the best possible physical fitness while effectively eliminating potential harmful factors and risky behaviours. These women were also involved in solving their own problems; they experienced real responsibility for their health and actions (manifestations in the form of behaviours—no addictions, professional involvement, developing interests, or acquiring the ability to adapt to changes).

In the group of men, the M_o1_ sub-model of responsibility for their own health was distinguished (see Figure 3).

The sub-model consisted of three men who refrained from driving fast. The respondents showed consistency of declarations regarding the criteria of health with actions undertaken in reality. They experienced a sense of responsibility for their health by undertaking, e.g., activities related to the development of healthy eating habits, or attempts to independently solve emerging problems related to the poor functioning of the body.

In the mixed group (including women and men), two sub-models (P_p1_, P_p2_) were distinguished, showing the transformation of responsibility for one’s health from apparent responsibility to real responsibility (see Figure 4).

### 3.4. P_p1_: People with Apparent Responsibility for Their Health

The sub-model consisted of fifty-six people who attended medical appointments irregularly, who did not follow recommendations or occasionally adjusted to certain beneficial and undemanding commitments, e.g., concerning diet. These people strongly believed in the possibility of returning to full strength after an illness thanks to other people, and declared they acknowledged numerous criteria of health but did not comply with any of them. The respondents also experienced a sense of guilt in the event of an illness.

### 3.5. P_p2_: People with Real Responsibility for Their Health

The sub-model consisted of twenty-six people who attended medical appointments with varying degrees of regularity. They followed dietary and medical recommendations and sought information on preventive measures to take to protect their health. They were aware of their impact on their well-being and had a positive attitude to life. They implemented most of the criteria of health they recognised, which lead them to improve the quality of their own lives and reduce harmful factors that may result in the deterioration of well-being. The respondents were also involved in building relationships with others at the family and social level.

A different approach to one’s health was presented by people who did not refrain from driving fast. The identified sub-models took into account personal characteristics, locus of control, criteria of health, and health involvement of young drivers.

In the group of women, two sub-models (W_n1_, W_n2_) were distinguished which showed the transformation of responsibility for one’s own health from excessively idealised responsibility to apparent responsibility (see Figure 5).

### 3.6. W_n1_: Women with Excessively Idealised Responsibility for Their Health

The sub-model consisted of twenty women who idealise their self-image (a responsible person, self-accepting, and easily adapting to changes) in order to gain social approval. They occasionally attended medical appointments. They were strongly convinced of the beneficial impact of the environment and happy coincidences on their well-being. It was true that they strongly idealised the criteria of health, but in practice, they did not implement them in everyday functioning.

### 3.7. W_n2_: Women with Apparent Responsibility for Their Health

The sub-model consisted of nineteen women who attempted to attend medical appointments when necessary. The respondents tried to follow medical recommendations, avoid tensions, and negative emotions. At the same time, they declared a hedonistic attitude to life. The respondents declared that in the event of health problems, they attended medical appointments and followed recommendations. However, these declarations were not followed by real actions. The women from this group were grateful to others for helping them fully recover. They created a positive (responsible, adaptive, and relatively coherent) self-image.

In the group of men, the M_n1_ sub-model was distinguished by men who are carefree and irresponsible with their health (see Figure 6).

### 3.8. M_n1_: Carefree Men, Irresponsible with Their Health

The sub-model consisted of eight men who did not refrain from driving fast and who displayed a hedonistic, trouble-free attitude to life. The respondents did not experience a sense of responsibility for their health and did not follow medical or dietary recommendations. In the event of an illness, they strongly believed in favourable circumstances and chance events that enabled them to return to full physical fitness. They declared they implement many criteria of health (e.g., problem-solving, adaptation to the situation, following dietary recommendations, or optimism in life); however, when confronted with a real health threat situation, they avoided taking any pro-health measures. They sought strong emotions and thrills, as additionally evidenced by their hedonistic attitude to life.

In the mixed group (including women and men), two sub-models (P_n1_, Pn_2_) were distinguished that showed the transformation of responsibility for one’s health from idealised responsibility to apparent responsibility (see Figure 7).

### 3.9. P_n1_: Persons with Idealised Responsibility for Their Health

The sub-model consisted of nineteen persons who sporadically attended medical appointments and who had difficulties in following any recommendations (medical, dietary). The respondents undertook real action related to avoiding unpleasant emotions, stresses and tensions. In their lives, they followed carefully selected and beneficial principles of healthy eating. These people were firmly convinced that recovery could be achieved thanks to other people and happy coincidences.

### 3.10. P_n2_: Personsw Apparent Responsibility for Their Health

The sub-model was made up of seventeen people who regularly attended medical appointments, but had difficulty complying with recommendations. They were positive about life; they avoided stress and tensions. They had a preference for strong emotional experiences. These people declared they were aware of the real impact on their health, but in the face of health threats, they were not consistent in undertaking health-promoting activities.

## 4. Discussion

Risky health behaviours are activities that can cause immediate or long-term negative health consequences [34]. They are sometimes considered undesirable and result in certain losses or harm to a person [35]. They are of interest to many researchers, as minimising them aims to improve health not only at the level of the individual but also of the entire population [36].

The analysis of the results obtained in this study shows that both understanding and control of health by young adults allow for predicting the tendencies for risky driving. The perception of health as a state is presented by Juczyński [15] in the questionnaire as *feeling happy most of the time*; *being able to enjoy life* or *feeling well* (p. 119). Health understood in this way may indicate a more hedonistic life orientation (focus on seeking pleasure “here and now”) [37,38]. Some researchers believe that hedonistic behaviours shape the contemporary lifestyle of young people [39]. Research by Butrym [40] shows that 41.2% of students when selecting their life orientation from given options, choose hedonism over eudaimonism. Unfortunately, this attitude is also reflected in their behaviours that have a negative impact on their health. They eat fast food more frequently, drink alcohol, take drugs, and psychoactive substances. Taking such actions also indicates a lower tendency to respect social norms. Research conducted by Zhu, Sze, N., and Bai [41] shows that social standards have a significant impact on the perception of road safety. Unfortunately, attitude towards one’s life and health also translates into risky driving.

The conducted analyses showed that the tendency to exceed speed limits occurs both in women and men who show the need to seek thrills and demonstrate the need to socially show off against other people [8]. A strong need for thrills, experiencing pleasure, and the culture of speed are undoubtedly becoming more and more frequently ubiquitous with many elements of the modern life of young people [42]. Interestingly, thrill-seekers tend to give less importance to risk. They are eager to anticipate risky situations in order to experience pleasure in them. The search for thrills is a tendency to consciously take risks, especially by young drivers [43]. In Bauman’s [44] analysis of various types of personality, one type he distinguishes is *the tourist*. Its characteristics partly correspond to the described behaviours of young adults prone to risky driving. According to this researcher, these people are characterised by a superficial demeanour, and involvement in the undertaken activities is limited to seeking only hedonistic sensations and experiences that young people collect. They are considered trophies and become an end in and of themselves. This could explain young adults engaging in risky driving as an activity that is an end by its very nature. This activity enriches the respondents with sensations derived from *here and now*, which make them feel happy. The obtained results are also consistent with the description of dangerous behaviours that belong to the *task-focused orientation* and were described by Wontorczyk [8]. The understanding of health control perceived as the impact of other significant people on the health of young adults was also a significant result of the present study.

The respondents were inclined to limit risky driving if the responsibility for their health was, to a greater extent, assigned to other people (doctors, nurses, teachers, or family) than to themselves. These results indicate the difficulty young adults have in taking responsibility for themselves and their health. The research by Bingham et al. [45] showed that the degree and frequency of risky driving behaviours decreased with increasing levels of psychosocial maturity. The analysis showed people who refrain from driving fast displayed responsibility for their health (taking specific actions in the event of health problems) and a sense of control over their lives. Based on the analyses, a clear coherence was found between pro-health activities and the criteria of health one recognises. Impulse control and decision-making skills are associated with the development of planning abilities and the formation of critical judgement [46,47]. These two elements are probably conducive to the processes of social and emotional maturation, significantly contributing to the decline in the tendency to engage in risky behaviours. It seems that the knowledge provided in the field of health should contribute to a better understanding of this value. However, as it turns out from the research by Stawarz et al. [14], in the opinion of academic youth, being healthy is understood differently depending on the person’s field of study. Particularly noteworthy is the fact that medical and technical students equate *being healthy* less with the fitness of *all parts of the body* than students of humanities. Stawarz et al. [14] indicate greater concerns of students of humanities than those of medical or technical students related to disability. At the same time, note that people studying medicine and technology have a lower need for cancer and cardiovascular disease prevention in relation to themselves. In the case of the results obtained in this article, young adults are coherent in their understanding of health and philosophy of life similar to the Epicurean phrase *carpe diem*.

Nosek et al. [48] cite various concepts of health in their work; however, they emphasise that it does not amount only to bio-psycho-social well-being, but it is also sometimes understood as a philosophy of life that directly affects health. An understanding of health similar to hedonistic views may be related to the developmental period in which young adults currently find themselves. Being beyond the control of their parents, they experience greater freedom and responsibility for the lifestyle they follow [16,49].

## 5. Conclusions

Summarizing the obtained results of our research, it can be concluded that:

(1) the adopted model of responsibility for health partly explains why young drivers engage in fast driving,
(2) the most important factor in the prediction of these risky behaviours is treating health as a state of pleasure,
(3) the transformation processes of the factors of responsibility for health are differentiated by gender:
  (a) the group that does not refrain from driving fast: for women they range from attitudes of idealising themselves and their abilities to have hedonistic attitudes, for men these are individuals with immediately hedonistic attitudes; a mixed group also emerged in which the initial attitudes are immediately characterised by pleasure tendencies that turn into a hedonistic attitude to life and thrill-seeking;
  (b) the group refraining from driving fast: for women transformations from avoidance attitudes to health-promoting behaviours, for men manifestations of responsibility for health, in the mixed group (from empty declarations to the integration of declarations with pro-health activities).

## Figures and Tables

**Table 1:** Descriptive statistics of socio-demographic variables.

Variables		Participants	
		*N*	%
Age	M	19.75	
	SD	1.11	
	Min	18.00	
	Max	30.00	
Sex	female	150.00	75.80
	male	48.00	24.20
Place of residence	RA	71.00	35.90
	T 50	17.00	8.60
	C 150	28.00	14.10
	LC400	45.00	22.70
Health condition	1.00	9.00	4.50
	2.00	12.00	6.10
	3.00	34.00	17.20
	4.00	100.00	50.50
	5.00	43.00	21.70
Awareness of the presence of chronic diseases in the family	Yes	64.00	32.30
	No	96.00	48.50
	I do not know	37.00	19.20

Note: RA—rural areas; T 50—Town with up to 50,000 inhabitants; C 150—City with up to 150,000 inhabitants; LC400—large cities with up to 400,000 inhabitants; 1—very bad; 2—bad; 3—average; 4—good; 5—very good.

**Table 2:** Values of regression coefficients affecting the driving style of young adults.

Predictors	B	SE	Wald	Exp (B)	*p*
Positive eating habits	−0.362	0.225	2.577	0.696	n.s.
Preventive behaviours	−0.162	0.249	0.426	0.850	n.s.
Positive psychological behaviours	0.317	0.285	1.230	1.372	n.s.
Health practices	−0.232	0.259	0.806	0.793	n.s.
Understanding health as a state	0.410	0.197	4.358	1.507	0.03
Understanding health as an objective	0.156	0.249	0.392	1.169	n.s.
Understanding health as a process	0.168	0.321	0.275	1.183	n.s.
Understanding health as a property	0.038	0.401	0.009	1.038	n.s.
Understanding health as an outcome	−0.124	0.341	0.133	0.883	n.s.
Internal health control	−0.027	0.032	0.720	0.973	n.s.
The effect of other people on my health	−0.066	0.030	4.723	0.936	0.03
The effect of chance on my health	0.032	0.029	1.242	1.033	n.s.
Gender (M)	−0.070	0.379	0.034	0.932	n.s.
Constant	1.683	1.470	1.311	5.382	n.s.
Nagelkerke’s R^2^ = 14.4; Hosmer-Lemeshow χ^2^ (8) = 4.07; *p* = 0.851					

## Data Availability

The data contained in this article has not been published in the repository but is available upon request.

## References

[1] Komenda Główna Policji Biuro Ruchu Drogowego Road Accidents in Poland in 2015–2018.

[2] Stawiarska-Lietzau M. (2011). Ryzykanci—Bohaterowie czy ofiary? Kulturowe uwarunkowania ryzykownej jazdy młodych mężczyzn. Prace Naukowe Akademii im. Jana Długosza Częstochowie Seria Pedagog..

[3] Riskfactors. https://www.obserwatoriumbrd.pl/pl/analizy_brd/problemy_brd/mlodzi_kierowcy/czynniki_ryzyka/.

[4] The Characteristics of the Group. https://www.obserwatoriumbrd.pl/pl/analizy_brd/problemy_brd/mlodzi_kierowcy/charakterystyka_grupy/.

[5] Who and Why Does Exceed the Speed Limits. https://www.obserwatoriumbrd.pl/pl/analizy_brd/problemy_brd/predkosc/kto-i-dlaczego-przekracza-predkosc/.

[6] Oviedo-Trespalacios O., Scott-Parker B. (2019). Fast and furious: A neglected issue in health promotion among young drivers. Health Promot. J. Aust. Off. J. Aust. Assoc. Health Promot. Prof..

[7] Jackowska B., Wycinka E. (2019). The influence of generation on the risk of road accidents. Statistical News. Pol. Stat..

[8] Wontorczyk A. (2011). Dangerous Behavior of Drivers. Psychological Model of Behavior Regulation in Road Traffic.

[9] Znajmiecka-Sikora M., Sałagacka M. (2018). Personal determinators of risk and aggressive behavior in the motorcycle group. Zesz. Nauk. Politech. Śląskiej. Seria Organ. Zarządzanie.

[10] Suchocka L. (2010). A Sense of Responsibility in Health and Disease. Noo-Theoretical Perspective.

[11] Wagner S., Banaszkiewicz M., Andruszkiewicz A., Strahl A., Miler A., Kubica A. (2015). Healthbehaviors and place of health in the hierarchy of youthvalues. Med. Ogólnai Nauk. Zdrowiu.

[12] Kulik A., Frańczyk E., Jazukiewicz I. (2018). Wisdom in the Hierarchy of Student Values in the Light of Empirical Research. Mądrość Jako Sprawność Moralna w Wychowaniu.

[13] Jaworski M., Adamus M., Bojar J. (2017). The Sense of Responsibility for the Health as an Important Element of Health Education. DEStech Trans. Soc. Sci. Educ. Hum. Sci..

[14] Stawarz B., Lewicka M., Sulima M., Wiktor M. (2014). Health as a value as assessed by students from the Podkarpackie Province. Ann. Acad. Med. Silesiensis.

[15] Juczynski Z. (2012). Measurementtools in Healthpromotion and Psychology.

[16] Sukys S., Cesnaitiene V.J., Ossowsky P.Z.W. (2017). Is Health Education at University Associated with Students’ Health Literacy?. BioMed Res. Int..

[17] Mazur J., Mazur J., Małkowska-Szkutnik A. (2018). Subjective health assessment. Health of Students in 2018 Against the Background of the New HBSC Research Model.

[18] Lally K., Nathan-V Y., Dunne S., McGrath D., Cullen W., Meagher D., Coffey J.C., Dunne C. (2015). Awareness of sexually transmitted infection and protection methods among university students in Ireland. Ir. J. Med. Sci..

[19] Burnett A.J., Sabato T.M., Walter K.O., Kerr D., Wagner L., Smith A. (2014). The Influence of Attributional Style on Substance Use and Risky Sexual Behavior Among College Students. Coll. Stud. J..

[20] Kawczyńska-Butrym Z., Butrym M., Czapka E. (2015). Students of Lublin universities. Health Risk Behaviors 20 Years Later.

[21] Andruszkiewicz A., Basińska M., Kubica A. (2010). Factorsinfluencing the level of motivation to quit smoking in the group of peopleaddicted to nicotine. Folia Cardiol. Excerpta.

[22] Ahmed F.M., Badawy R.E. (2016). Health Locus of Control and Health Promoting Behaviors among Nursing and Non-Nursing Students in Zagazig University. IOSR J. Nurs. Health Sci..

[23] Pirzadeh A., Moghaddam E.S., Araghinezhad Z.E., Ardakani T., Torkian S. (2019). Health Locus of Control among Students of Isfahan University of Medical Sciences (2018). Health Educ. Health Promot..

[24] Cohen M., Azaiza F. (2007). Health-promoting behaviors and health locus of control from a multicultural perspective. Ethn. Dis..

[25] Helmer S.M., Krämer A., Mikolajczyk R.T. (2012). Health-related locus of control and health behaviour among university students in North Rhine Westphalia, Germany. BMC Res. Notes.

[26] Figner B., Mackinlay R.J., Wilkening F., Weber E.U. (2009). Affective and deliberative processes in risky choice: Age differences in risk taking in the columbia card task. J. Exp. Psychol. Learn. Mem. Cogn..

[27] Willoughby T., Good M., Adachi P.J., Hamza C., Tavernier R. (2013). Examining the link between adolescent brain development and risk taking from asocial–developmental perspective. Brain Cogn..

[28] Rzechowska E. (2014). 50+ workers at risk of redundancy, or what counsellors should know: A psychological perspective. J. Couns..

[29] Brandmaier A.M., von Oertzen T., McArdle J.J., Lindenberger U. (2013). Structural equation model trees. Psychol. Methods.

[30] Hand D., Manilla H., Smyth P. (2001). Principles of Data Mining.

[31] Wu X., Kumar V., Quinlan J.R., Ghosh J., Yang Q., Motoda H., McLachlan G.J., Ng A., Liu B., Yu P.S. (2008). Top 10 algorithms in data mining. Knowl. Inf. Syst..

[32] An Example of Forecasting Using Data Mining Methods. StatSoft Polska. https://www.statsoft.pl/Portals/0/Downloads/Przyklad_prognozowania.pdf.

[33] Quinlan J.R. (1993). C4.5: Programs for Machine Learning.

[34] Ay S., Yanıkkerem E., Çalim S., Yazici M. (2012). Health-promoting lifestyle behaviour for cancer prevention: A survey of Turkish university students. Asian Pac. J. Cancer Prev. APJCP.

[35] Cairns R.B., Cairns B.D. (1994). Lifelines and Risks: Pathways of Yout in Our Time.

[36] Henson J.M., Carey M.P., Carey K.B., Maisto S.A. (2006). Associations among health behaviors and time perspective in young adults: Model testing with boot-strapping replication. J. Behav. Med..

[37] Hamzah S.R., Suandi T., Krauss S.E., Hamzah A., Tamam E. (2014). Youth hedonistic behaviour: Moderating role of peer attachment on the effect of religiosity and worldview. Int. J. Adolesc. Youth.

[38] Kanclerz B. (2015). Life Orien- tations of Youth—Conditions and Changes]. Studia Eduk..

[39] Thorpe H. (2012). Sex, drugs and snowboarding: Legitimate definitions of taste and lifestyle in a physical youth culture. Leis. Stud..

[40] Butrym M., Krawczyńska-Butrym Z., Butrym M., Czapska E. (2015). Pleasure or renunciation—Determinants of behaviorrelated to healthrisk. Students of Lublin Universities. Health Risk Behaviors 20 Years Later.

[41] Zhu D., Sze N.N., Bai L. (2021). Roles of personal and environmental factors in the red light running propensity of pedestrian: Case study at the urban crosswalks. Transp. Res. Part F Traffic Psychol. Behav..

[42] Graham H.M., White R.D. (2007). Young people, dangerous driving and car culture. Youth Stud. Aust..

[43] Jessor R. (1987). Risky driving and adolescent problem behaviour: An extension of problem-behavior theory. Alcohol Drugs Driv..

[44] Bauman Z. (2011). Post modern personalpatterns. Studia Socjol..

[45] Bingham C.R., Shope J.T., Zakrajsek J., Raghunathan T.E. (2008). Problem driving behaviour and psychosocial maturation in young adulthood. Accid. Anal. Prev..

[46] Giedd J.N. (2004). Structural magnetic resonance imaging of the adolescent brain. Ann. N. Y. Acad. Sci..

[47] Chen T., Sze N.N., Saxena S., Pinjari A.R., Bhat C.R., Bai L. (2020). Evaluation of penalty and enforcement strategies to combat speeding offences among professional drivers: A Hong Kong stated preference experiment. Accid. Anal. Prev..

[48] Nosek M.A., Hughes R.B., Howland C.A., Young M.E., Mullen P.D., Shelton M.L. (2004). The meaning of health for women with physical disabilities: A qualitative analysis. Fam. Community Health.

[49] Von Ah D., Ebert S., Ngamvitroj A., Park N., Kang D.H. (2004). Predictors of health behaviours in college students. J. Adv. Nurs..

